# Supplementary figures and images for: Nup93 regulates breast tumor growth by modulating cell proliferation and actin cytoskeleton remodeling
Source: Life Sci Alliance. 2020 Jan 20;3(1):e201900623. doi: 10.26508/lsa.201900623 (PMC6971368; doi:10.26508/lsa.201900623)

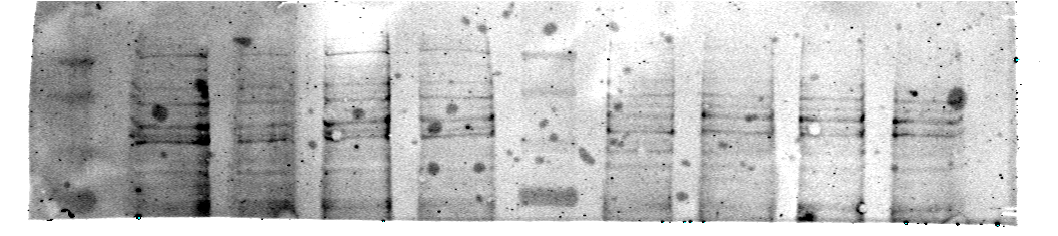

Supplement: Supplementary file 3 [file LSA-2019-00623_SdataF2_1.tif]
